# Longitudinal associations between TV viewing and BMI not explained by the ‘mindless eating’ or ‘physical activity displacement’ hypotheses among adults

**DOI:** 10.1186/s12889-018-5674-4

**Published:** 2018-06-26

**Authors:** Verity J. Cleland, Kira Patterson, Monique Breslin, Michael D. Schmidt, Terence Dwyer, Alison J. Venn

**Affiliations:** 1 Menzies Institute for Medical Research, University of Tasmania, Private Bag 23, Hobart, TAS 7001 Australia; 2 Faculty of Education, University of Tasmania, Launceston, TAS Australia; 3 The George Institute for Global Health, University of Oxford, Oxford, England; 4 Department of Kinesiology, University of Georgia, Athens, USA

**Keywords:** Diet, Food and nutrition, Body weights and measures, Behaviour, Health, Health promotion

## Abstract

**Background:**

The mechanisms explaining the positive relationship between television (TV) viewing and body mass index (BMI) are unclear. ‘Mindless eating’ and ‘physical activity displacement’ theories have been suggested, but have not been tested longitudinally among young adults. This study aimed to determine whether longitudinal associations between young adults’ TV viewing and BMI are explained by changes in TV-related food and beverage consumption (FBC) and/or leisure-time physical activity (LTPA) over 5 years among young adults.

**Methods:**

A cohort of young Australian adults (*n* = 1068) was assessed in 2004–6 (T1) and 2009–2011 (T2), height and weight were measured (T1) or self-reported (T2), and participants self-reported TV viewing time (hours/day), weekly TV-related FBC and LTPA (mins/week). Linear regression was used to examine direct pathways between TV viewing and BMI, adjusting for TV-related FBC and LTPA to examine indirect pathways.

**Results:**

The association between TV viewing time and BMI (β: 0.41, 95% CI 0.03, 0.78 for > 1-h increase in TV viewing/day) was not explained by TV-related FBC (β: 0.37, 95% CI -0.18, 0.91) or LTPA (β: 0.38, 95% CI -0.17, 0.93) hypotheses. Increased TV-related FBC was associated with increased TV viewing (0.39 ± 1.54 h/day) and greater increases in BMI (0.92 ± 2.28 kg/m^2^, *p* = 0.16). LTPA increases were not associated with changes in TV viewing (− 0.07 ± 1.42 h/day), and increases in BMI were smallest when LTPA increased (0.44 ± 2.25 kg/m^2^) and greatest when LTPA decreased (0.82 ± 2.30 kg/m^2^) (*p* = 0.13).

**Conclusions:**

Factors other than changes in TV-related FBC or LTPA may explain the longitudinal relationship between TV viewing and increasing BMI among young adults. Findings confirm that TV viewing is a risk factor for weight gain in young adults but the underlying causal mechanisms remain unclear.

**Electronic supplementary material:**

The online version of this article (10.1186/s12889-018-5674-4) contains supplementary material, which is available to authorized users.

## Background

Sedentary behaviours such as sitting are estimated to increase the relative risk of type 2 diabetes (by 12%), cardiovascular events (by 147%), cardiovascular mortality (90%) and all-cause mortality (49%) [1, 2]. These associations are largely independent of moderate- to vigorous-intensity physical activity. [1, 2] Television (TV) viewing is a discretionary and modifiable sedentary behaviour that is prevalent in developed countries such as the United States and Australia, with adults spending around 2–3 h per day watching TV [3, 4]. This is alarming, because for each 2 hours per day of TV viewing, the relative risks of type 2 diabetes, cardiovascular disease and all-cause mortality increases by 13–20% [5]. TV viewing therefore offers an important behavioural target for interventions that may contribute to improvements in population health.

Evidence from prospective observational studies [6, 7] suggests that greater TV viewing is directly associated with higher risk of overweight and obesity, higher body mass index (BMI) and larger waist circumference values, and that more TV viewing leads to increased adiposity [8]. There are a number of potential explanations for the association between TV viewing and adiposity, but two commonly cited are the ‘displacement’ and ‘mindless eating’ theories [9]. Displacement theory suggests that TV viewing displaces time that could be spent in other more active pursuits, but the largely cross-sectional evidence to support this notion [10–13] has come under scrutiny in recent times with an increased focus on the association between sedentary behaviours and adiposity, independent of moderate- to vigorous-intensity physical activity [8, 14–16].

The ‘mindless eating’ hypothesis purports that food consumption during TV viewing increases overall energy intake, leading to weight gain. Observational studies in children have identified associations between TV viewing time and intake of energy dense foods overall [17–19], and an intervention found children consumed more energy when the TV was turned on than when switched off [20]. However, very few studies have examined the impact on adiposity of food and beverage consumption while watching TV among adults.

There is some support for the ‘mindless eating’ hypothesis from a small pilot intervention study [21] and a large cross-sectional observational study [22]. However, no longitudinal studies have attempted to disentangle the role of TV-related food and beverage consumption and physical activity. This study aimed to determine whether associations between TV viewing time and BMI were explained by changes in TV-related food and beverage consumption and/or by leisure-time physical activity. We hypothesised that longitudinal associations between TV viewing time and BMI would be partially explained by changes in TV-related food and beverage consumption (a broad indicator of ‘mindless eating’) and leisure-time physical activity (a broad indicator of ‘displacement’). We use a five-year longitudinal observational study of young adults to investigate this hypothesis, a study sample chosen for its unique combination of measures of TV viewing, physical activity, TV-related food and beverage consumption and BMI, and prospective design.

## Methods

We report using the STROBE guidelines for observational studies (Additional file 1).

### Study population

**Fig. 1:**
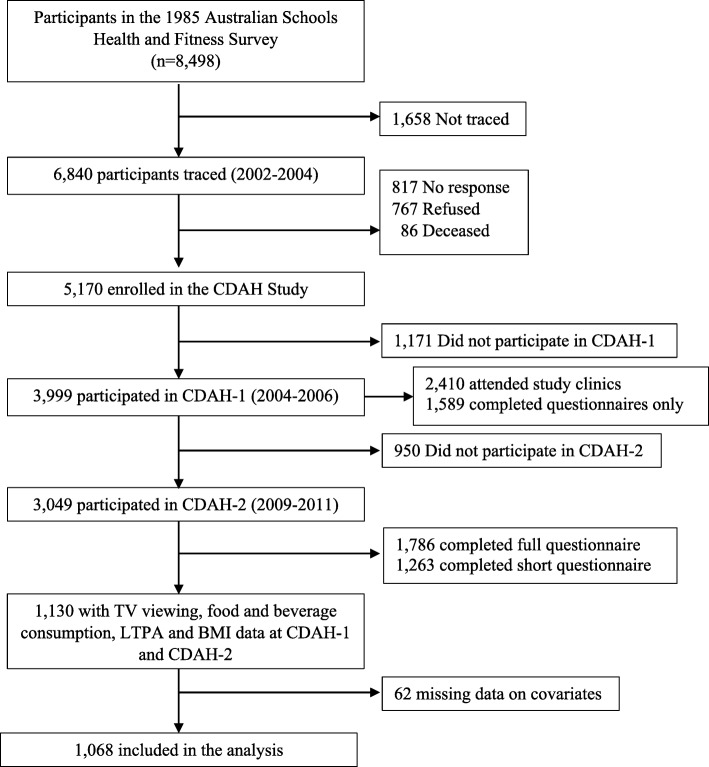
Flowchart of participation

Data were derived from the 20- and 25-year follow-up studies of the 1985 Australian Schools Health and Fitness Survey (ASHFS), [23, 24] known as CDAH1 (Childhood Determinants of Adult Health study, 2004–6) and CDAH2 (Childhood Determinants of Adult Health study, 2009–10) [24, 25] (Fig. 1). Participants were eligible for the CDAH studies if they had participated in the 1985 ASHFS. The ASHFS included a nationally representative sample of 8498 Australian school children aged 7–15 years from 109 public (government), Catholic and independent (private) schools. Limited tracking information was collected in 1985, but participants were found by searching online directories, historical and current electoral rolls, school networks and contact with enrolled participants. Response proportions and loss to follow-up for the CDAH studies have been reported elsewhere [26], but in brief, 2410 adults completed questionnaires and attended a 3-h study clinic in CDAH1 (aged 26–36 years) with a further 1589 completing questionnaires only, while 1786 adults (aged 31–41 years) completed full questionnaires in CDAH2 (an abbreviated shortened version was offered to those who refused to complete the full set of questionnaires in order to maximise participation; this shortened version did not collect information on all of the variables required for this analysis). Of these, 1068 participants met the inclusion criteria for this paper (see *Statistics*, below).

### Measures

#### BMI and weight status

In CDAH1, BMI (kg/m^2^) was calculated from measured height and weight, while in CDAH2, height and weight were self-reported. A correction factor was developed using a linear regression model of data from participants whose weight and height was both self-reported and measured [27]. Weight status was classified as healthy (BMI < 25 kg/m^2^), overweight (BMI 25 kg–29.9 kg/m^2^) or obese (BMI ≥30 kg/m^2^). Change in BMI was calculated by subtracting BMI at CDAH1 from BMI at CDAH2.

#### TV viewing

In both CDAH1 and CDAH2, participants reported total TV viewing in the past week (including DVDs and videos) when this was the main activity they were doing, with separate totals for weekdays and weekend days [28]. Change in hours/day TV viewing time was calculated by subtracting TV time at CDAH1 from TV time at CDAH2. A three-level categorical change variable was also created with change between CDAH1 and CDAH2 classified as stable, ≥1-h increase, and ≥ 1-h decrease. These categories were selected as they provide practically relevant units of time.

#### TV-related food and beverage consumption

In CDAH1 and CDAH2, participants reported separately how often they consumed a meal, snack, soft drink or alcoholic drink during TV viewing. Response options were always (every day), usually (5–6 times/week), sometimes (3–4 times/week), rarely (1–2 times/week) or never. A summary ‘total TV-related food and beverage consumption’ variable was created for participants using responses from each item, which was classified as high (≥5 times/wk. consumes ≥1 item), medium (3–4 times/wk. consumes ≥1 item) or low (≤2 times/wk. consumes all four items). Change in total consumption was classified as ‘no change’ (low at both time points, medium at both time points, or high at both time points), ‘increased’ (moved from low at CDAH1 to medium or high at CDAH2, or from medium at CDAH1 to high at CDAH2) or ‘decreased’ (moved from high or medium at CDAH1 to low at CDAH2, or from medium at CDAH1 to low at CDAH2). A similar change variable was created for each of the individual TV-related food and beverage consumption behaviours (meals, snacks, soft drinks, alcoholic beverages), similarly classified as ‘no change’, ‘increased’ and ‘decreased’.

#### Leisure-time physical activity (LTPA)

In CDAH1 and CDAH2, participants reported past week moderate- to vigorous-intensity LTPA (activities done solely for recreation, sport, exercise or leisure) as part of the long version of the International Physical Activity Questionnaire [29]. Change in LTPA was calculated by subtracting LTPA at CDAH1 from LTPA at CDAH2. We also developed a variable to represent change in LTPA categorically as stable, > 1-h increase, > 1-h decrease. These categories were selected as they provide practically relevant units of time.

### Statistics

To assess change between CDAH1 and CDAH2 in sociodemographic characteristics, outcome, exposure and potential mediator variables, we used the Stuart-Maxwell test [30, 31] for testing differences in dependent samples (categorical variables) and paired t-tests (continuous variables).

**Fig. 2:**
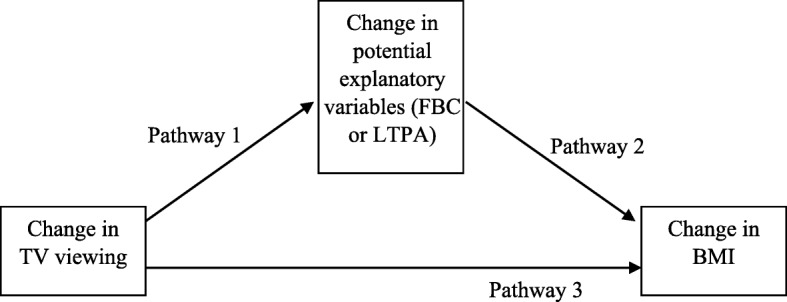
Potential pathways to explain relationships between television (TV) viewing, BMI, TV-related food and beverage consumption (FBC) and leisure-time physical activity (LTPA)

Pathways between change in TV viewing and change in BMI were examined according to the pathway diagram in Fig. 2. The association between change in TV viewing and the potential mediators (Pathway 1) was tested using one-way analysis of variance (ANOVA) (categorical TV-related food and beverage consumption and LTPA variables) and Spearman correlation coefficients (continuous change in LTPA). Association between the potential mediators and change in BMI (Pathway 2) similarly was tested using ANOVA (categorical change in total TV-related food and beverage consumption and LTPA variables) and Spearman correlation coefficients (continuous change in LTPA). Linear regression models were used to look for evidence of mediation of the effect of change in TV viewing through change in food and beverage consumption, and through change in LTPA. A model with TV change predicting BMI change, adjusted for confounders, was compared with models including the hypothesised mediators. Any indirect effect through the mediators would be apparent in a reduction in the coefficient for change in TV viewing.

We tested for interactions between gender, change in TV viewing and BMI; none were identified. Self-reported age, highest level of education, marital status, employment status, occupation, number of children, and current smoking were considered as potential confounders in the final models; those that resulted in > 10% change in the coefficients were included.

Analyses were restricted to the sample with complete data for required covariates, and contained 1068 individuals. The approach taken to address bias resulting from attrition from the original random sample of children was to create inverse propensity weights [32] to model probability of response. Any observations required for the propensity model that were missing were imputed using multiple imputation by chained equations [33], so that a complete set of weights for the analysis sample was available. Fifty different datasets were imputed, and for each dataset a set of weights was derived and applied to the analysis model. The resulting 50 different model estimates were combined using Rubin’s rules [33] to get an average point estimate and standard error that reflected the variation in the weights. A second strategy to explore the impact of loss to follow-up was to compare our sample to the Australian population.

All analyses were performed in Stata Version 12 (Statacorp, College Station, Texas).

## Results

Approximately half of the participants were university-educated and around half were in managerial or professional occupations (Table 1). The proportion of married participants, the number of participants with one or more children, BMI and overweight and obesity all increased over the 5-year follow-up period. Significantly fewer women were employed in a full-time capacity at CDAH2 compared with CDAH1, and fewer participants were smokers at CDAH2. Participants watched on average just under 2 h of TV per day (similar to the national average of 1.89 h/day [3]) and did just over 2.5 h of LTPA each week, with no evidence of change over time. High overall consumption of food during TV time decreased significantly, falling to around 50% at CDAH2.

**Table 1:** Characteristics of the Childhood Determinants of Adult Health study sample in (2004–6 and 2009–11)

Characteristics	All			Men			Women		
	CDAH-1 (*n* = 1068)	CDAH-2 (*n* = 1068)	p^a^	CDAH-1 (*n* = 384)	CDAH-2 (*n* = 384)	p^a^	CDAH-1 (*n* = 684)	CDAH-2 (*n* = 684)	p^a^
Potential Confounders									
Age (years), M (SD)	31.5 (2.6)	36.4 (2.6)	< 0.001	31.7 (2.6)	36.7 (2.5)	< 0.001	31.4 (2.6)	36.3 (2.6)	< 0.001
Education, *n* (%)									
University	555 (52.0)	579 (54.2)		189 (49.2)	198 (51.6)		366 (53.5)	381 (55.7)	
Vocational/diploma/trade	268 (25.1)	292 (27.3)		116 (30.2)	128 (33.3)		152 (22.2)	164 (24.0)	
School only	245 (22.9)	197 (18.5)	0.001	79 (20.6)	58 (15.1)	< 0.001	166 (24.3)	139 (20.3)	< 0.001
Occupation, *n* (%)									
Manager or professional	608 (56.9)	628 (58.8)		258 (67.2)	275 (71.6)		350 (51.2)	353 (51.6)	
White collar	204 (19.1)	198 (18.5)		29 (7.6)	27 (7.0)		175 (25.6)	171 (25.0)	
Blue collar	109 (10.2)	100 (09.4)		85 (22.1)	71 (18.5)		24 (3.5)	29 (4.2)	
Not in labour force	147 (13.8)	142 (13.3)	0.37	12 (3.1)	11 (2.9)	0.03	135 (19.7)	131 (19.2)	0.18
Marital status, n (%)									
Single	254 (23.8)	154 (14.4)		112 (29.2)	54 (14.1)		142 (20.8)	100 (14.6)	
Married or living as married	782 (73.2)	872 (81.7)		265 (69.0)	320 (83.3)		517 (75.6)	552 (80.7)	
Separated or divorced	31 (2.9)	42 (3.9)	< 0.001	7 (1.8)	10 (2.6)	< 0.001	24 (3.5)	32 (4.7)	< 0.001
Number of children^b^, n (%)									
0	324 (47.4)	308 (28.8)		–	126 (32.8)		324 (47.4)	182 (26.6)	
1	137 (20.0)	184 (17.2)		–	72 (18.8)		137 (20.0)	112 (16.4)	
2	160 (23.4)	375 (35.1)		–	133 (34.6)		160 (23.4)	242 (35.4)	
≥ 3	63 (9.2)	201 (18.8)	–	–	53 (13.8)		63 (9.2)	148 (21.6)	< 0.001
Employment status, *n* (%)									
Full-time	686 (64.2)	588 (55.1)		350 (91.2)	354 (92.2)		336 (49.1)	234 (34.2)	
Part-time	197 (18.5)	313 (29.3)		20 (5.2)	13 (3.4)		177 (25.9)	300 (43.9)	
Other	185 (17.3)	167 (15.6)	< 0.001	14 (3.7)	17 (4.4)	0.14	171 (25.0)	150 (21.9)	< 0.001
Smoking status, *n* (%)									
Non-smoker	867 (81.2)	925 (86.6)		307 (80.0)	327 (85.2)		560 (81.9)	598 (87.4)	
Smoker	201 (18.8)	143 (13.4)	< 0.001	77 (20.1)	57 (14.8)	0.14	124 (18.1)	86 (12.6)	< 0.001
Outcome Variables									
BMI (kg/m^2^), M(SD)	25.2 (4.9)	25.9 (5.2)	< 0.001	26.1 (4.2)	26.7 (4.6)	< 0.001	24.7 (5.2)	25.4 (5.5)	< 0.001
Weight status, n (%)									
Healthy weight	598 (56.0)	538 (50.4)		167 (43.5)	143 (37.2)		431 (63.0)	395 (57.8)	
Overweight	327 (30.6)	347 (32.5)		159 (41.4)	173 (45.1)		168 (24.6)	174 (25.4)	
Obese	143 (13.4)	183 (17.1)	< 0.001	58 (15.1)	68 (17.7)	< 0.001	85 (12.4)	115 (16.8)	< 0.001
Exposure Variable									
TV (hrs/day), Med (IQR)	1.7 (1.7)	1.6 (1.6)	0.77	2.0 (1.8)	2.0 (1.7)	0.58	2.0 (1.8)	1.6 (1.4)	0.35
Explanatory Variables									
LTPA (mins/wk), Med (IQR)	101 (240)	120 (220)	0.96	120 (239)	101 (240)	0.23	100 (195)	120 (210)	0.30
Total TV-related FBC^c^, n (%)									
Low	183 (17.1)	231 (21.6)		43 (11.2)	231 (21.6)		140 (20.5)	164 (24.0)	
Medium	284 (26.6)	313 (29.3)		100 (26.0)	313 (29.3)		184 (26.9)	204 (29.8)	
High	601 (56.3)	524 (49.1)	< 0.001	241 (62.8)	524 (49.1)	0.001	360 (52.6)	316 (46.2)	0.002
TV-related meals, n (%)									
0 times/wk	129 (12.1)	194 (18.2)		34 (8.9)	194 (18.2)		95 (13.9)	130 (19.0)	
1–2 times/wk	228 (21.4)	268 (25.1)		73 (19.0)	268 (25.1)		155 (22.7)	178 (26.0)	
3–4 times/wk	239 (22.4)	215 (20.1)		80 (20.8)	215 (20.1)		159 (23.3)	137 (20.0)	
≥ 5 times/wk	472 (44.2)	391 (36.6)	< 0.001	197 (51.3)	391 (36.6)	< 0.001	275 (40.2)	239 (34.9)	< 0.001
TV-related snacks, *n* (%)									
0 times/wk	113 (10.6)	127 (11.9)		32 (8.3)	127 (11.9)		81 (11.8)	85 (12.4)	
1–2 times/wk	385 (36.1)	378 (35.4)		126 (32.8)	378 (35.4)		259 (37.9)	258 (37.7)	
3–4 times/wk	322 (30.2)	330 (30.9)		124 (32.3)	330 (30.9)		198 (29.0)	205 (30.0)	
≥ 5 times/wk	248 (23.2)	233 (21.8)	0.69	102 (26.6)	233 (21.8)	0.47	146 (21.4)	136 (19.9)	0.97
TV-related soft drinks, *n* (%)									
0 times/wk	515 (48.2)	608 (56.9)		154 (40.1)	608 (56.9)		361 (52.8)	419 (61.3)	
1–2 times/wk	314 (29.4)	265 (24.8)		125 (32.6)	265 (24.8)		189 (27.6)	160 (23.4)	
3–4 times/wk	140 (13.1)	119 (11.1)		57 (14.8)	119 (11.1)		83 (12.1)	63 (9.2)	
≥ 5 times/wk	99 (9.3)	76 (7.1)	< 0.001	48 (12.5)	76 (7.1)	0.001	51 (7.5)	42 (6.1)	< 0.001
TV-related alcohol, n (%)									
0 times/wk	472 (44.2)	467 (43.7)		117 (30.5)	467 (43.7)		355 (51.9)	333 (48.7)	
1–2 times/wk	413 (38.7)	388 (36.3)		165 (43.0)	388 (36.3)		248 (36.3)	245 (35.8)	
3–4 times/wk	126 (11.8)	149 (14.0)		68 (17.7)	149 (14.0)		58 (8.5)	72 (10.5)	
≥ 5 times/wk	57 (5.3)	64 (6.0)	0.41	34 (8.9)	64 (6.0)	0.06	23 (3.4)	34 (5.0)	0.40

^a^Determined by Stuart-Maxwell test for categorical variables and paired t-tests for continuous variables for differences between CDAH1 and CDAH2 overall and for men and women

^b^Data on number of children were not collected at CDAH1 among men

^c^Classified as high (≥5 times/wk. consumes ≥1 item), medium (3–4 times/wk. consumes ≥1 item) or low (≤2 times/wk. consumes all four items)

CDAH1 Childhood Determinants of Adult Health study (2004–06); CDAH2 Childhood Determinants of Adult Health study (2009–10); LTPA leisure-time physical activity, *TV* television, *FBC* food and beverage consumption, *Med* median, *IQR* inter-quartile range

While there was no evidence of change in average TV viewing hours/week over time, categorical measures identified around 20% of participants who increased their TV viewing by more than 1 h/day, and 20% who decreased their TV viewing by more than 1 h/day (Table 2). One third of participants increased their LTPA by more than 1 h/day, 28% decreased their LTPA by more than 1 h/day, and 39% demonstrated no change in LTPA. The proportion of participants who decreased their TV-related food and beverage consumption ranged from 20.2% (alcohol) to 33% (meals), and the proportion of participants who increased their TV-related food and beverage consumption ranged from 15.1% (soft drinks) to 27.2% (snacks). No change was evident among 42% (snacks) to 57% (soft drinks and alcohol) of participants.

**Table 2:** Change in television viewing, physical activity and television-related food and beverage consumption (2004–6 to 2009–11)

Categorical change variables	Total (*n* = 1068)	Men (*n* = 384)	Women (*n* = 684)
Change in TV viewing (hrs/day), n (%)			
Stable	643 (60.2)	213 (55.5)	430 (62.9)
> 1 h increase	209 (19.6)	88 (22.9)	121 (17.7)
> 1 h decrease	216 (20.2)	83 (21.6)	133 (19.4)
Change in LTPA, n (%)			
Stable	418 (39.1)	152 (39.6)	266 (38.9)
> 1 h increase	353 (33.1)	113 (29.4)	240 (35.1)
> 1 h decrease	297 (27.8)	119 (31.0)	178 (26.0)
Change in total TV-related FBC, n (%)			
No change	589 (55.2)	214 (55.7)	375 (54.8)
Increased	188 (17.6)	61 (15.9)	127 (18.6)
Decreased	291 (27.3)	109 (28.4)	182 (26.6)
Change in TV-related meals, n (%)			
No change	506 (47.4)	180 (46.9)	326 (47.7)
Increased	210 (19.7)	68 (17.7)	142 (20.8)
Decreased	352 (33.0)	136 (35.4)	216 (31.6)
Change in TV-related snacking, n (%)			
No change	451 (42.2)	158 (41.2)	293 (42.8)
Increased	290 (27.2)	104 (27.1)	186 (27.2)
Decreased	327 (30.6)	122 (31.8)	205 (30.0)
Change in TV-related soft drinks, n (%)			
No change	614 (57.5)	205 (53.4)	409 (59.8)
Increased	161 (15.1)	64 (16.7)	97 (14.2)
Decreased	293 (27.4)	115 (30.0)	178 (26.0)
Change in TV-related alcohol, n (%)			
No change	611 (57.2)	212 (55.2)	399 (58.3)
Increased	241 (22.6)	79 (20.6)	162 (23.7)
Decreased	216 (20.2)	93 (24.2)	123 (18.0)

CDAH1 Childhood Determinants of Adult Health study (2004–06); CDAH2 Childhood

Determinants of Adult Health study (2009–10); LTPA leisure-time physical activity, *TV* television, *FBC* food and beverage consumption

### Pathway 1: TV viewing and potential explanatory variables

Positive associations were observed between change in TV viewing time and change in all TV-related food and beverage consumption variables (except alcohol) (Table 3). There was no association between change in TV viewing time and change in LTPA (categorically or continuously; Spearman correlation coefficient − 0.02, *p* = 0.58).

**Table 3:** Change in television viewing and BMI by change in explanatory variables from baseline to follow-up

Change in potential explanatory variables	Participants	Pathway 1: Change in TV viewing from CDAH-1 to CDAH-2	Pathway 2: Change in BMI from CDAH-1 to CDAH-2
	n	hours/day	Kg/m^2^
Change in total FBC during TV viewing			
No change	589	0.02 (1.43)	0.54 (2.39)
Increased	188	0.39 (1.54)	0.92 (2.28)
Decreased	291	−0.34 (1.42)	0.65 (2.50)
*P* value^2^		< 0.001	0.16
Change in TV-related meal consumption			
No change	506	0.03 (1.47)	0.64 (2.31)
Increased	210	0.24 (1.52)	0.81 (2.63)
Decreased	352	−0.23 (1.39)	0.53 (2.39)
*P* value^2^		< 0.001	0.42
Change in TV-related snack consumption			
No change	451	−0.03 (1.52)	0.53 (2.41)
Increased	290	0.31 (1.42)	0.90 (2.15)
Decreased	327	−0.27 (1.39)	0.56 (2.59)
*P* value^2^		< 0.001	0.09
Change in TV-related soft drink consumption			
No change	614	0.00 (1.30)	0.59 (2.32)
Increased	161	0.39 (1.61)	0.90 (2.54)
Decreased	293	−0.26 (1.65)	0.60 (2.50)
*P* value^2^		< 0.001	0.32
Change in TV-related alcohol consumption			
No change	611	0.00 (1.34)	0.71 (2.31)
Increased	241	0.01 (1.60)	0.70 (2.34)
Decreased	216	−0.07 (1.65)	0.36 (2.69)
*P* value^2^		0.79	0.16
Change in LTPA, n (%)			
Stable	418	0.05 (1.53)	0.67 (2.58)
> 1 h increase	353	−0.07 (1.42)	0.44 (2.25)
> 1 h decrease	297	−0.04 (1.43)	0.82 (2.30)
*P* value^2^		0.54	0.13

^1^Mean; standard deviations in parentheses (all such values)

^2^Determined by one-way ANOVA

CDAH1 Childhood Determinants of Adult Health study (2004–06); CDAH2 Childhood Determinants of Adult Health study (2009–10); *LTPA* leisure-time physical activity, *TV* television, *FBC* food and beverage consumption

### Pathway 2: Potential explanatory variables and BMI

There was no statistically significant association between change in TV-related food and beverage consumption behaviour and change in BMI (Table 3), with BMI increasing in all groups. However, there was a trend for BMI increases to be higher among those who increased their TV-related food and beverage consumption behaviours or decreased LTPA, and to be lower amongst those who decreased their TV-related food and beverage consumption or increased their LTPA. Change in BMI and continuous change in LTPA were weakly inversely correlated (Spearman correlation coefficient − 0.10, *p* = 0.002); those whose BMI remained stable or increased demonstrated a median change in LTPA of 0 mins/week, while those whose BMI decreased had a median increase in LTPA of 30 mins/week.

### Pathway 3: TV viewing and BMI

Continuous measures of change in TV viewing demonstrated a weak positive association with change in BMI, adjusted for age, sex and education (Table 4). Those whose TV viewing increased by more than 1 h/day demonstrated a BMI at CDAH2 0.41 kg/m^2^ (95% CI 0.03, 0.78) greater than those whose TV viewing remained stable (adjusted for CDAH1 BMI); this association was marginally attenuated but became non-significant (0.38 kg/m^2^, 95% CI -0.18, 0.94) after applying sample weights to account for loss to follow-up. Nearly identical results were observed when we used change in BMI between CDAH-1 and CDAH-2 as the outcome (data not presented).

**Table 4:** Association^1^ between change in television viewing from baseline to follow-up and BMI (*n* = 1068) (Pathway 3)

Change in daily TV viewing	BMI at CDAH-2 adjusted for BMI at CDAH-1	
	Unweighted sample β (95% CI)	Weighted sample^2^ β (95% CI)
Change in TV viewing (hrs/day)	0.08 (− 0.01, 0.18)	0.09 (− 0.04, 0.22)
Categories of change in TV viewing		
Stable		
> 1 h increase	0.41 (0.03, 0.78)^3^	0.38 (− 0.18, 0.94)
> 1 h decrease	−0.02 (− 0.40, 0.35)	0.04 (− 0.46, 0.53)

^1^Adjusted for age, sex, education

^2^Inverse probability weights were created from the comprehensive data collected over three time points. Observations that were missing were imputed using multiple imputation by chained equations so that a complete set of weights for the analysis sample was available; we imputed 50 different datasets, and for each dataset a set of weights was derived and applied to the analysis model. The final estimate is the average of these model estimates, along with confidence intervals derived using Rubin’s rules (26)

^3^Significantly different from reference category in linear regression analyses

CDAH1 Childhood Determinants of Adult Health study (2004–06); CDAH2 Childhood Determinants of Adult Health study (2009–10); TV television

### Mediating variables and the association between TV viewing and BMI

Neither TV-related food and beverage consumption (Model 2), LTPA (Model 3) or TV-related food and beverage consumption and LTPA combined (Model 4) explained the association between change in TV viewing and BMI at CDAH-2 (Table 5). As can be seen, the difference in beta coefficients between Model 1 (adjusted for age, sex, education and BMI) and the other models (adjusted for TV-related food and beverage consumption, LTPA, or both) was minimal (0.01–0.02), providing no evidence of mediation. No difference in the findings were noted when change in BMI between CDAH1 and CDAH2 was used as the outcome (data not shown), or when each of the individual TV-related food and beverage consumption behaviours were added to the model separately (data not presented).

**Table 5:** Association between change in television viewing and BMI (weighted associations^1^)

Daily TV viewing (hrs/day)	BMI at CDAH2 (adjusted for BMI at CDAH1) B (95% CI)
Model 1 (M1)	Age, sex, education
Stable	(ref)
> 1 h increase	0.38 (− 0.17, 0.93)
> 1 h decrease	0.01 (− 0.48, 0.49)
Model 2 (M2)	M1 + Overall food & beverage consumption
Stable	(ref)
> 1 h increase	0.37 (− 0.18, 0.91)
> 1 h decrease	0.02 (− 0.47, 0.50)
Model 3 (M3)	M1 + LTPA
Stable	(ref)
> 1 h increase	0.38 (− 0.17, 0.93)
> 1 h decrease	0.01 (− 0.47, 0.50)
Model 4 (M4)	M1 + M2 + M3
Stable	(ref)
> 1 h increase	0.36 (− 0.19, 0.91)
> 1 h decrease	0.02 (− 0.46, 0.51)

^1^Inverse probability weights were created from the comprehensive data collected over three time points. Observations that were missing were imputed using multiple imputation by chained equations so that a complete set of weights for the analysis sample was available; we imputed 50 different datasets, and for each a set of weights were derived and applied to the analysis model. The average of these model estimates was used to derive a set of weights for the analysis model

CDAH1 Childhood Determinants of Adult Health study (2004–06); CDAH2 Childhood Determinants of Adult Health study (2009–10); TV television

## Discussion

We found little evidence that longitudinal associations between TV viewing time and BMI are explained by either TV-related food and beverage consumption (‘mindless eating’) or leisure-time physical activity (‘displacement’) among young adults. This is the first study to attempt to disentangle the complex longitudinal pathways between TV viewing, LTPA, TV-related food and beverage consumption, and BMI during early- to mid-adulthood. Although a clear and direct association was evident between changes in TV viewing and changes in TV-related food and beverage consumption, this behaviour did not translate to an explanation of the relationship between TV viewing and BMI over time. Changes in moderate- to vigorous-intensity LTPA were not associated with changes in TV viewing or with changes in BMI, providing little support for the displacement hypothesis.

This lack of support for the displacement hypothesis supports some [8, 14–16] recent evidence that has highlighted the independent nature of sedentary behaviour and physical activity in associations with adiposity. While not unanimous [10–13], there is growing evidence that the impact of sedentary behaviour on a range of outcomes, including all-cause mortality, is largely independent of moderate- to vigorous-intensity physical activity [34], and that these behaviours should be targeted separately in public health interventions. While the evidence-base for the sedentary behaviour-adiposity relationship is not as well-established as that for these other outcomes, the current study reinforces that support for the displacement theory is diminishing.

That only a weak association of borderline statistical significance between TV viewing and BMI was detected is consistent with much of the existing evidence from prospective studies. Evidence from three systematic reviews [35–37] and one review of systematic reviews [38] fails to demonstrate a strong prospective relationship between sedentary behaviour and weight/adiposity among adults. This may be due to reasons such as the time lag between fluctuations in behaviour and weight change, lack of prospective studies with three or more time points, residual confounding, or lack of precision in the measures used.

There are a number of potential explanations for the finding that TV-related food and beverage consumption did not explain the TV viewing-BMI relationship, such as there being other more important mechanisms at play. For instance, TV advertisements for nutrient-poor, calorie-dense foods, the use of targeted product placements in TV shows, the influence on social perceptions of body image, and TV programs that portray cooking, eating and losing weight as entertainment may stimulate food intake at other times of the day, encourage overeating, or establish unrealistic behavioural or weight loss expectations [9]. While most of these alternative mechanisms were not assessed in this study, we were able to conduct a sensitivity analysis to examine the impact of change in ‘extra food’ consumption and change in a dietary guideline index. We found these variables also did not explain the relationship between TV viewing and BMI.

It is also possible that the findings observed here are related to measurement error in the outcome or explanatory variables. Although we have previously demonstrated cross-sectional associations between TV-related food and beverage consumption and abdominal adiposity [22], it is possible that the measure of TV-related food and beverage consumption was not sensitive enough to detect changes in behaviour over time. Data collected were limited to the frequency of consumption, and not the quantities or types (e.g. diet vs. non-diet soft drinks) consumed, which may reduce sensitivity to detect associations. In spite of these limitations, this and our earlier cross-sectional work demonstrate consistent associations with adiposity. Another possible reason for our findings is that general dietary behaviours may account for the relationship between TV viewing and obesity. For example, higher levels of TV viewing may be associated with a poorer diet overall, and not necessarily reflect eating behaviours that occur during TV viewing. While there is evidence to suggest that overall diet is associated with TV viewing among children and adolescents [39], evidence among adults is sparse. While screen-based activities other than TV viewing such as smartphone or tablet use are increasing, TV viewing time has remained relatively stable and still accounts for a large proportion of adults’ screen time [40, 41]. Future studies need to consider the use of modern screen-based technologies in their assessments.

BMI was calculated from measured weight and height in CDAH-1, but from self-reported weight at CDAH-2, introducing the possibility of measurement error. To help address this, we applied a correction factor generated from participants with both measured and self-reported height. Given that weight is typically under-reported, the likely impact on our findings is an under-estimation of the associations. Physical activity and TV viewing were measured via self-report, and although reliable [28] and valid [29] instruments were used, it is still possible that there was misreporting of physical activity or TV viewing, which may impact on the ability to detect associations. However, in a sensitivity analysis of a sub-sample of 734 participants who wore Yamax pedometers at both time points, similar null effects were seen, suggesting that misreporting may not be a valid explanation. Further, we restricted our focus to activities performed during leisure time only, as appropriate for examination of the ‘displacement’ hypothesis (i.e., we would not hypothesise TV viewing to displace work-related physical activity). However, it is possible that TV viewing displaces activities performed in other domains, for instance, domestic activity or transport-related activity (although these were likely captured by our pedometer measures), or that TV viewing displaces light-intensity activity, which was not measured. Little change was evident in our continuous measures of LTPA which may also have impacted on our ability to explain associations; however, we categorised LTPA to demonstrate that for a sizeable proportion of our sample (around 60%), changes of important magnitude (> or < 1 h) did occur. While differences were not statistically significant, those who increased their LTPA had substantially smaller increases in BMI (approximately half) than those who decreased their LTPA.

Data were drawn from a prospective cohort study of children who were followed up in adulthood 20- and 25-years later, with substantial loss to follow-up between childhood and the first adult follow-up, and further loss between the first and second adult follow-ups. This analysis focused on the two adult follow-ups, and using the extensive baseline information available for all participants, we were able to comprehensively characterise participants and non-participants and use multiple imputation and inverse probability weighting techniques to reduce the likelihood of bias, providing greater confidence in the generalisability and external validity of our findings. There was no difference in the main outcome (BMI) between CDAH1 participants who did and did not participate in CDAH2 (25.2 vs 25.7 kg/m2). There were also no differences in TV-related food and beverage consumption, age or weight, but there were some differences in other variables. Those who participated only in CDAH1 were more commonly male (47% vs. 36%), watched less TV (2.0 vs. 2.9 h/day), had lower levels of education (27% not completing year 12 vs 23% not completing), smoked more (24% current smokers vs 19% current smokers), and were marginally more physically active (171 vs 161 mins/week LTPA). All of these variables were amongst those included in the model for propensity weights.

Participants in this study were similar to national Australian adult data in terms of TV viewing time (2.0 h/day for men and 1.8 h/day for women in CDAH vs 1.89 h/day nationally [3]), but a higher proportion of CDAH2 participants were married and/or living as married (82% vs. 61%), were employed as professionals and/or managers (59% vs. 31%), and were university educated (54% vs. 31%) [42, 43]. Other strengths include the prospective five-year follow-up, our ability to examine the effects of a range of potential confounding factors, and a focus on an important target group for preventive efforts – young adults – who experience significant life stage transitions (e.g., partnering, parenting) that have the potential to impact positively (e.g. reduced alcohol consumption) and negatively on obesity-risk behaviours [44, 45].

## Conclusion

In conclusion, the modest longitudinal relationship we observed between TV viewing and BMI was not explained by changes in either TV-related food and beverage consumption or changes in LTPA. Although broad indicators of ‘mindless eating’ and ‘displacement’, these findings provide little support for these hypotheses as explanations for the TV viewing-obesity relationship. However, changes in TV viewing were strongly related to changes in TV-related food and beverage consumption, reinforcing the importance of public health messages to discourage TV viewing. This is the first longitudinal study to attempt to disentangle the complex interplay between TV viewing, LTPA, TV-related food and beverage consumption and weight. While there were some limitations that may have impacted on findings (likely resulting in an under-estimate of effects), we know of no other longitudinal cohorts that have tested these important hypotheses in a large sample of young adults. This work provides some longitudinal evidence that TV viewing is a risk factor for weight gain in young adults, but the underlying causal mechanisms remain unclear. Future longitudinal studies would benefit from the use of dietary measures that allow better assessment of diet quality and energy density to help understand the basis for the relationship between dietary behaviours and TV viewing. Further longitudinal work is to establish causality, and to identify other possible mechanisms explaining this relationship.

## Additional file

Additional file 1: STROBE Statement—checklist of items that should be included in reports of observational studies. (DOC 110 kb)

## Abbreviations

ASHFS: Australian Schools Health and Fitness Survey
BMI: Body mass index
CDAH1: Childhood Determinants of Adult Health study (2004–6)
CDAH2: Childhood Determinants of Adult Health study (2009–10)
FBC: Food and beverage consumption
LTPA: Leisure-time physical activity
TV: Television
