# Supplementary material for: Longitudinal associations between TV viewing and BMI not explained by the ‘mindless eating’ or ‘physical activity displacement’ hypotheses among adults
Source: BMC Public Health. 2018 Jun 26;18:797. doi: 10.1186/s12889-018-5674-4 (PMC6019267; doi:10.1186/s12889-018-5674-4)
Supplement: Supplementary file 1 — STROBE Statement—checklist of items that should be included in reports of observational studies. (DOC 110 kb) [file 12889_2018_5674_MOESM1_ESM.doc]

**STROBE Statement—checklist of items that should be included in reports of observational studies**

|  | Item No | Recommendation | Page + line number in manuscript |  |  |
| --- | --- | --- | --- | --- | --- |
| **Title and abstract** | 1 | (*a*) Indicate the study’s design with a commonly used term in the title or the abstract | Page 1, line 2 |  |  |
|  |  | (*b*) Provide in the abstract an informative and balanced summary of what was done and what was found | Page 3 |  |  |
| Introduction | | |  |  |  |
| Background/rationale | 2 | Explain the scientific background and rationale for the investigation being reported | Pages 5-6 |  |  |
| Objectives | 3 | State specific objectives, including any prespecified hypotheses | Page 6, lines 128-133 |  |  |
| Methods | | |  |  |  |
| Study design | 4 | Present key elements of study design early in the paper | Page 6 |  |  |
| Setting | 5 | Describe the setting, locations, and relevant dates, including periods of recruitment, exposure, follow-up, and data collection | Page 6-8, lines 137-179 |  |  |
| Participants | 6 | (*a*) *Cohort study*—Give the eligibility criteria, and the sources and methods of selection of participants. Describe methods of follow-up  *Case-control study*—Give the eligibility criteria, and the sources and methods of case ascertainment and control selection. Give the rationale for the choice of cases and controls  *Cross-sectional study*—Give the eligibility criteria, and the sources and methods of selection of participants |  |  |  |
|  |  | (*b*) *Cohort study*—For matched studies, give matching criteria and number of exposed and unexposed  *Case-control study*—For matched studies, give matching criteria and the number of controls per case | n/a |  |  |
| Variables | 7 | Clearly define all outcomes, exposures, predictors, potential confounders, and effect modifiers. Give diagnostic criteria, if applicable | Pages 7-8, lines 152-182; page 9-10, lines 199-222 |  |  |
| Data sources/ measurement | 8* | For each variable of interest, give sources of data and details of methods of assessment (measurement). Describe comparability of assessment methods if there is more than one group | Pages 7-8, lines 152-182 |  |  |
| Bias | 9 | Describe any efforts to address potential sources of bias | Page 10 and 15, line 225 and 349 |  |  |
| Study size | 10 | Explain how the study size was arrived at | Page 6-7, lines 140-149 |  |  |
| Quantitative variables | 11 | Explain how quantitative variables were handled in the analyses. If applicable, describe which groupings were chosen and why | Pages 8-10, lines 190-232 |  |  |
| Statistical methods | 12 | (*a*) Describe all statistical methods, including those used to control for confounding | Pages 9-10, lines 194-235 |  |  |
|  |  | (*b*) Describe any methods used to examine subgroups and interactions | Pages 9-10, lines 194-235 |  |  |
|  |  | (*c*) Explain how missing data were addressed | Page 10, lines 228-230 |  |  |
|  |  | (*d*) *Cohort study*—If applicable, explain how loss to follow-up was addressed  *Case-control study*—If applicable, explain how matching of cases and controls was addressed  *Cross-sectional study*—If applicable, describe analytical methods taking account of sampling strategy | Page 10, lines 224-235 |  |  |
|  |  | (*e*) Describe any sensitivity analyses | Pages 13 and 14, lines 311-315 and 332-333 |  |  |

| Results | | |  | | | |
| --- | --- | --- | --- | --- | --- | --- |
| Participants | 13* | (a) Report numbers of individuals at each stage of study—eg numbers potentially eligible, examined for eligibility, confirmed eligible, included in the study, completing follow-up, and analysed | Page 6-7, lines 137-149 |  |  | |
|  |  | (b) Give reasons for non-participation at each stage |  |  |  | |
|  |  | (c) Consider use of a flow diagram | Page 6-7, lines 137-149; Figure 1 |  |  | |
| Descriptive data | 14* | (a) Give characteristics of study participants (eg demographic, clinical, social) and information on exposures and potential confounders | Page 21, Table 1 |  |  | |
|  |  | (b) Indicate number of participants with missing data for each variable of interest | Page 6-7, lines 141-149 |  |  | |
|  |  | (c) *Cohort study*—Summarise follow-up time (eg, average and total amount) | Page 6, lines 137-140 |  |  | |
| Outcome data | 15* | *Cohort study*—Report numbers of outcome events or summary measures over time | Pages 10-12, lines 238- 292, Tables 1-5 |  |  | |
|  |  | *Case-control study—*Report numbers in each exposure category, or summary measures of exposure | N/A |  |  | |
|  |  | *Cross-sectional study—*Report numbers of outcome events or summary measures | N/A |  |  | |
| Main results | 16 | (*a*) Give unadjusted estimates and, if applicable, confounder-adjusted estimates and their precision (eg, 95% confidence interval). Make clear which confounders were adjusted for and why they were included | Page 9, lines 199-222; Table 5 |  |  | |
|  |  | (*b*) Report category boundaries when continuous variables were categorized | Table 2-3 |  |  | |
|  |  | (*c*) If relevant, consider translating estimates of relative risk into absolute risk for a meaningful time period | N/A |  |  | |
| Other analyses | 17 | Report other analyses done—eg analyses of subgroups and interactions, and sensitivity analyses | Page 10, lines 224-235; page 12, lines 286-292; page 13, lines 316-320; page 14, lines 336-353 |  |  | |
| Discussion | | |  |  |  |  |
| Key results | 18 | Summarise key results with reference to study objectives | Pages 13-16 |  |  | |
| Limitations | 19 | Discuss limitations of the study, taking into account sources of potential bias or imprecision. Discuss both direction and magnitude of any potential bias | Pages 13-16 |  |  | |
| Interpretation | 20 | Give a cautious overall interpretation of results considering objectives, limitations, multiplicity of analyses, results from similar studies, and other relevant evidence | Pages 13-16 |  |  | |
| Generalisability | 21 | Discuss the generalisability (external validity) of the study results | Page 15, lines 349-360 |  |  | |
| Other information | | |  |  |  |  |
| Funding | 22 | Give the source of funding and the role of the funders for the present study and, if applicable, for the original study on which the present article is based | Page 2, lines 26-33 |  |  | |
|  |  |  |  |  |  | |

*Give information separately for cases and controls in case-control studies and, if applicable, for exposed and unexposed groups in cohort and cross-sectional studies.

**Note:** An Explanation and Elaboration article discusses each checklist item and gives methodological background and published examples of transparent reporting. The STROBE checklist is best used in conjunction with this article (freely available on the Web sites of PLoS Medicine at http://www.plosmedicine.org/, Annals of Internal Medicine at http://www.annals.org/, and Epidemiology at http://www.epidem.com/). Information on the STROBE Initiative is available at www.strobe-statement.org.
